# Sediment Characterization at the Equatorial Mid‐Atlantic Ridge From P‐to‐S Teleseismic Phase Conversions Recorded on the PI‐LAB Experiment

**DOI:** 10.1029/2018GL080565

**Published:** 2018-11-23

**Authors:** M. R. Agius, N. Harmon, C. A. Rychert, S. Tharimena, J.‐M. Kendall

**Affiliations:** ^1^ Ocean and Earth Science University of Southampton Southampton UK; ^2^ Now at the Department of Geosciences Faculty of Science, University of Malta Msida Malta; ^3^ Now at Jet Propulsion Laboratory California Institute of Technology Pasadena CA USA; ^4^ School of Earth Sciences University of Bristol Bristol UK

**Keywords:** PI‐LAB, sediments, equatorial Mid‐Atlantic Ridge

## Abstract

Accurate marine sediment characteristics, for example, thickness and seismic velocity, are important for constraining sedimentation rates with implications for climate variations and for seismic imaging of deeper structures using ocean bottom seismic deployments. We analyze P‐to‐S seismic phase conversions from the sediment‐crust boundary recorded by the Passive Imaging of the Lithosphere‐Asthenosphere Boundary (PI‐LAB) experiment to infer the sediment thickness across the Mid‐Atlantic Ridge covering 0‐ to 80‐Myr‐old seafloor. We find P_d_ s‐P delay times of 0.04–0.37 s, or 5‐ to 82‐m thickness. Sediment thickness increases with age. Thickness agrees with global estimates for young (<15–20 Myr) seafloor but is thinner on older lithosphere. Our result may represent a lower limit on sediment thickness, given that several of our stations are on topographic highs. The sedimentation rate decrease observed from 5 to 1.2 mm/kyr at ∼10 Myr suggests a recent increase in productivity related to climate change, eolian dust fluxes, and/or biogenic marine activity.

## Key Points

- P‐to‐S delay times from the sediment‐crust boundary suggest 5‐82 m of sediment beneath PI‐LAB
- The thickness generally agrees with global estimates but is less than expected on older lithosphere
- Higher deposition rates at <10 Myr suggest higher productivity perhaps related to stronger monsoons

## 1 Introduction

In the deep ocean basins away from continental shelves, sedimentation is composed of biogenic matter, which is a function of local productivity, a terrestrial component due to aerosols and local submarine weathering and erosion. Aerosol and biogenic productivity are a function of the climate and ocean temperature through time and so are important for our understanding of the paleoclimate and oceans. In addition, accurate sediment characteristics (e.g., thickness and seismic velocity) are important for accurately imaging the oceanic crust and mantle. Our understanding of these sediments and sedimentation rates are based on the analysis of core samples extracted from drilling into the ocean seafloor (e.g., Ocean Drilling Program), active source seismic studies (e.g., Mehouachi & Singh, 2018; Seher et al., 2010), and passive source seismic studies (e.g., Harmon et al., 2007; Lewis & Dorman, 1998; Shearer & Orcutt, 1987; Ruan et al., 2014; Rychert et al., 2018). Some of these data are also used to compile a global model of sediment thickness (Whittaker et al., 2013), which incorporates crustal age and distance from the continents. Drilling and active seismic sources provide high resolution but are usually limited in areal extent. Passive seismic deployments offer the opportunity to constrain sediment thickness over a larger area and range of seafloor age.

**Figure 1:**
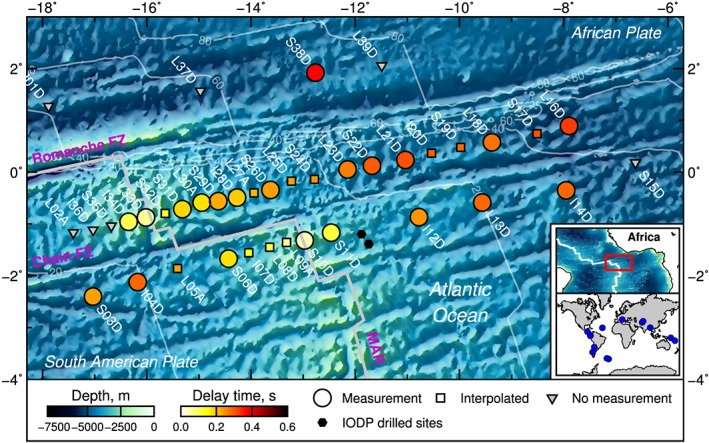
P s sediment delay time across the Mid‐Atlantic Ridge (MAR). Circles are average measurements, squares are linear interpolated measurements from the two nearest neighbors along the same SW‐NE profile, and triangles are stations without data, where no interpolation is applied; together they make the Passive Imaging of the Lithosphere‐Asthenosphere Boundary seismic network. Black hexagons: International Ocean Drilling Project (IODP) drilled holes Leg 108 sites 662 and 663 (Ruddiman & Janecek, 1989). Thick gray line indicates the ridge (Bird, 2003). Thin contours: Seafloor age at 20‐Myr intervals (Müller et al., 2008). Inset maps: Location of the study area (red box) on a regional map and the earthquakes used in this study (blue dots).

The Passive Imaging of the Lithosphere‐Asthenosphere Boundary (PI‐LAB) experiment presents a unique opportunity to examine deep ocean basin scale sedimentation. There has been some drilling along the Mid‐Atlantic Ridge (MAR; e.g., Karson et al., 1997; Kelemen et al., 2007) with a few sites close to the equator (Figure 1; Ruddiman & Janecek, 1989), although overall the region is sparsely sampled. PI‐LAB network consisted of 39 ocean bottom seismometers deployed at the equatorial Mid‐Atlantic Ocean on 0‐ to 80‐Myr seafloor from March 2016 to March 2017. The stations were broadband 120‐s period (station names starting with letter L) and 240‐s period (S and I), deployed on both sides of the ridge and centered on the Chain Fracture Zone (Figure 1). Here we use the P‐to‐S seismic phase conversions from the sediment‐crust boundary to estimate the sediment velocity and thickness beneath each station and infer the sedimentation history of the region.

## 2 Thickness Estimation

**Figure 2:**
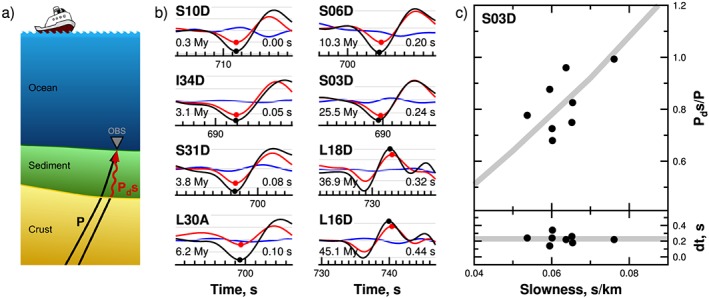
Examples of Ps sediment conversions, and relationship of P_d_ s/P amplitude ratio and delay time (P_d_ s‐P, dt) with respect to slowness. (a) Schematic of the raypaths of the P wave and P‐to‐S conversion from the base of the sediment layer, black and red arrows, respectively. (b) Black and red waveforms show the direct P wave and the converted S wave on the vertical and radial component, respectively. Note that there is no incoming signal on the transverse component (blue seismogram). The maximum absolute amplitude of the P phase and the following S phase are marked with dots. The seafloor age and delay time are indicated in the bottom left and right, respectively. Each frame is the record from a different station for the same teleseismic earthquake. (c) Top: P_d_ s/P amplitude ratio with respect to slowness for earthquakes recorded at station S03D. Gray line: P_d_ s/P ratio from synthetic waveforms using a 1‐D model and a range of ray parameters. Bottom: Delay time with respect to slowness for earthquakes recorded at station S03D. Horizontal gray line: Average Ps delay time for the station. Note that dt remains stable for a wide range of ray parameters. OBS = ocean bottom seismometer.

Sediments have a sharp seismic velocity and density contrast to the underlying basaltic crust. Upward propagating compressional (*P*) waves are converted at the boundary between the sediment and the crust to shear (*S*) waves (*P*_*d*_ *s*), which are both recorded on the ocean bottom seismometers (Figure 2a). The delay time (*dt*) between the two phases (*P*_*d*_ *s*‐*P*) and their relative amplitude depend on thickness and seismic velocities of the sedimentary layer. Here we characterize sediments using teleseismic earthquakes recorded by the PI‐LAB experiment, with analysis similar to works by Harmon et al. (2007) and Rychert et al. (2018).

We processed the seismic records in the following way. First, we removed the instrument response. We then determined the station horizontal orientations using *P* wave polarization (Magotra et al., 1989) and Rayleigh wave polarization (Doran & Laske, 2017). Both methods gave horizontal orientations that were within error of each other. The seismic records of teleseismic events with near‐vertical raypaths were rotated into the radial and transverse components using the orientation from *P* wave. We demeaned the data, and band‐pass filtered between 0.04 and 0.25 Hz. Teleseismic earthquakes that exhibited clear *P* wave arrival on the vertical component and clear *P*‐to‐*S* converted phase from the sediment‐crust interface on the radial component were chosen by visual inspection. A window one wavelength long from the *P* wave arrival is selected. The timing of the maximum absolute amplitude of the *P* phase and the following *P*_*d*_ *s* phase peak were then determined automatically within the window. Figure 2b shows examples of *P* and *P*_*d*_ *s* converted phases at different stations for the same earthquake (24 November 2016, 18:43 UTC, Mw 7.0). The delay time between the two phases is less than 0.5 s, and measurements are robust independent of earthquake magnitude or distance (Figure 2c, bottom frame).

The filtering is necessary to avoid picking secondary peaks in the waveforms due to noise in the data; however, special attention is taken to ensure that delay times, and in turn the sediment thickness, are not underestimated due to the choice of filter (e.g., Ritsema et al., 2009). The delay time could be underestimated at low frequency if the *P* wave and the *P*‐to‐*S* conversion interfere, which can occur for nonvertical incidence. We do not expect large degrees of interference because the incidence angles of *P* waves in slow sediment is small, typically <8°. Nevertheless, we also performed a test in which we analyzed the data using a high‐pass filter 0.04–20 Hz. There is no systematic bias and the results are in good agreement regardless of filter (supporting information Figure S1). In another test, we determine the accuracy of the sediment delay time from the converted phase given a realistic crustal structure (supporting information Figure S2). We find that difference between the predicted and the measured delay times is small, well within errors. The reason that the filter does not affect our results is because the sediment is thin, and very slow, and the waves are traveling nearly vertically so there is no interference between the *P* and the *P*‐to‐*S* conversion.

The sediment thickness is inferred using previously determined relationships between delay time and seismic velocities of compressional waves (*V*_*P*_; e.g., Nafe & Drake, 1957) and shear waves (*V*_*S*_; e.g., Ruan et al., 2014). The empirical function of Ruan et al. (2014), is given by

$$V_{S}(h)=(ah^{2}+\mathrm{bh}+cV_{0})/(h+c)$$

where *V*_0_ is the wave speed of the sediment at the seafloor, 0.1 km/s (e.g., Hamilton, 1979), *h* is the depth below the seafloor, and *a*, *b*, and *c* are three constant parameters 0.15608, 1.2198, and 0.49473, respectively (Bell et al., 2015). These parameters were established by inverting Rayleigh wave ratios of vertical displacement‐to‐pressure for sediment thicknesses and shear velocity at the seafloor of the Juan de Fuca Ridge (Bell et al., 2015; Ruan et al., 2014).

The empirical relationship of Nafe and Drake (1957) for deep water is given by

$$V_{P}=0.43h+1.83$$

The thickness is calculated from the delay time via the following equation:

$$dt=h(\sqrt{{\left(1/V_{S}\right)}^{2}-u^{2}}-\sqrt{{\left(1/V_{P}\right)}^{2}-u^{2}})$$

where *u* is the ray parameter.

We verify this relationship is valid at one example station, S03D, which recorded a large number of waveforms. We compared the observed amplitudes and delay times of the *P*‐to‐*S* conversions with those from synthetic seismograms (Shearer & Orcutt, 1987) computed assuming the predicted sediment shear velocity (*V*_*S*_=0.14 km/s) from the previously determined relationship developed for pelagic sediment in Cascadia using compliance (Ruan et al., 2014), and *V*_*P*_=1.84 km/s from reflection (Nafe & Drake, 1957; Figure 2). We assumed crustal *P* wave velocities increase from 4.5 to 7.5 km/s from the surface to the Moho adapted from seismic refraction measurements in the north MAR (Seher et al., 2010). The predicted amplitudes show good agreement with our observations (Figure 2c).

We further tested this assumption by determining our own best fitting parameters (sediment *V*_*P*_, sediment *V*_*S*_, and sediment thickness) at example station S03D. We compared the amplitude and delay time of synthetic seismograms to our observed values using a grid search approach searching over *V*_*P*_ and *V*_*S*_ (supporting information Figure S3). We find that the values of Ruan et al. (2014) and Nafe and Drake (1957), used here, are within 1*σ* confidence of the grid search. Similar range of *V*_*S*_ values have been obtained from in situ measurements (Hamilton, 1976) and nonlinear waveform fitting (Nolet & Dorman, 1996; *V*_*S*_=0.31–0.37 km/s at 100‐m thick sediments). Lower values for *V*_*P*_ (∼1.55 km/s) have been obtained from nearby cores drilled by the International Ocean Drilling Project (IODP sites 662 and 663; Figure 1; Shipboard Scientific Party, 1988a, 1988b). However, assuming this *V*_*P*_ results in minimal changes in sediment thickness (supporting information Figure S3). Similarly, other relationships for *V*_*P*_ thickness relationships would have little effect on our results, given the similarity of many other relationships to that of Nafe and Drake (1957; e.g., Hamilton, 1979) and also that we have little sensitivity to *V*_*P*_ velocity‐thickness relationships (Ruan et al., 2014).

Of course thickness and velocity trade‐off. However, our tests at S03D suggest that the previously determined velocity‐thickness relationships are a good match. In addition, the gird search test at S03D suggests that, if anything, the thickness could be lower than that determined using the previously derived relationships. We did not record a sufficient number of usable waveforms to allow us to determine our own sediment thickness‐shear wave velocity relationship at the other stations in the array because the uncertainty is too high.

**Figure 3:**
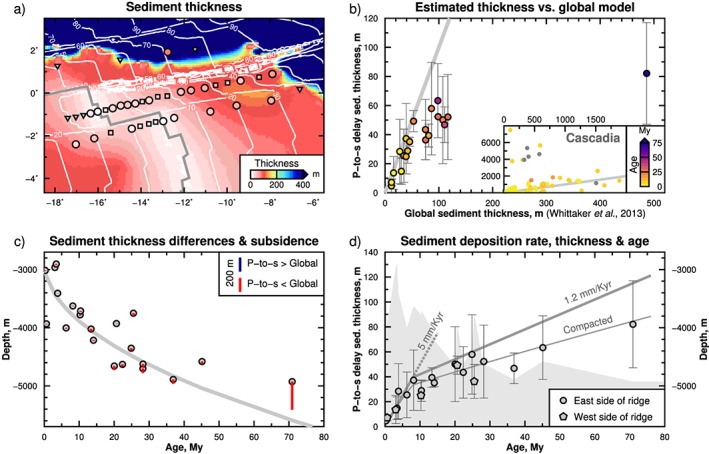
Comparison of estimated sediment thickness with global sediment model, age, subsidence, and production rate. (a) Estimated sediment thickness overlaid on the global sediment model of the world's oceans (Whittaker et al., 2013). Symbols are the same as in Figure 1. (b) Comparison of P‐to‐S delay sediment thickness with the global model. Error bars show the uncertainty. Inset: Comparison of estimated sediment thickness with the global model for Cascadia (Rychert et al., 2018). Gray line: One‐to‐one relationship between the estimated and global model. Different colors represent the seafloor age below the station. Gray dots: Stations without an age constraint. (c) Difference in sediment thickness between estimated and global model (vertical bars), and regional subsidence determined by binning and averaging the acquired bathymetry data (Harmon et al., 2018; $\mathrm{depth}=2861+324.3\times \sqrt{\mathrm{age}}$). (d) Sediment deposition rate, thickness, and seafloor age. Thick gray line: Estimated accumulated sediment thickness over time at the indicated deposition rates. Thin gray line: Inferred compacted sediment thickness (Alibés et al., 1996). Gray background: The depth at each station. Pentagon and circles: Stations located on west and east side of the ridge, respectively.

In total, we measured 86 *P*‐to‐*S* conversions from 16 earthquakes with magnitude >6.0 Mw, recorded on 21 stations. Sediment properties are interpolated to station locations that did not yield a reliable result so that they may be better accounted for in future seismic imaging work. The linear interpolation is between the two adjacent stations along the same SW‐NE profiles (Figures 1 and  3a). We did not interpolate to stations that did not record data or were not recovered. The average delay times, sediment thicknesses, standard errors, and interpolated values of all the stations are listed in Table 1 and shown in Figure 3. The error in our estimated sediment thickness is relatively large in comparison to the thicknesses themselves, sometimes over 50%. However, this is expected given that thicknesses are very small and also the frequency content of our waveforms. Despite this error, we observe increasing thickness with age, as expected, and a clear age‐thickness trend in the data, again suggesting that the result is robust.

**Table 1:** PI‐LAB Stations Longitude, Latitude, Elevation, Average P s Sediment Delay Time, Number of Events Used, Standard Error, Average Sediment Thickness, and Error

Station	Longitude	Latitude	Elevation (m)	Average delay (s)	NoE	Error (s)	Average thickness (m)	Error (m)
I01D	−17.8855	1.2734	−4,047					
L02A	−17.4085	−1.1667	−3,499					
S03D	−17.0315	−2.4021	−3,750	0.23	8	0.06	36	13.61
I04D	−16.1733	−2.1238	−3,928	0.29	3	0.03	49	7.27
L05A	−15.4100	−1.8600	−4,052	0.24^a^			38^a^	
S06D	−14.4298	−1.6703	−3,778	0.17	6	0.06	25	12.01
I07D	−14.0428	−1.5565	−3,819	0.14^a^			20^a^	
L08D	−13.6409	−1.4493	−3,357	0.10^a^			14^a^	
L09A	−13.3185	−1.3569	−3,378	0.07^a^			9^a^	
S10D	−12.9697	−1.3180	−3,015	0.04	4	0.04	5	4.35
S11D	−12.4602	−1.1691	−2,905	0.11	5	0.06	15	9.04
I12D	−10.7766	−0.8683	−4,022	0.25	2	0.03	39	7.84
L13D	−9.5619	−0.5862	−4,659	0.28	2	0.11	50	30.03
I14D	−7.9524	−0.3522	−4,702	0.28	3	0.12	52	29.28
S15D	−6.6228	0.1814	−4,927					
L16D	−7.8953	0.8933	−4,581	0.33	6	0.07	63	25.50
S17D	−8.5121	0.7422	−5,205	0.31^a^			56^a^	
L18D	−9.3765	0.5769	−4,890	0.28	5	0.05	47	12.28
S19D	−9.9754	0.4809	−4,607	0.28^a^			49^a^	
I20D	−10.5352	0.3681	−4,724	0.29^a^			51^a^	
L21D	−11.0380	0.2364	−4,625	0.30	1		52	
S22D	−11.6799	0.1254	−4,352	0.30	11	0.12	58	31.84
L23D	−12.1478	0.0521	−4,631	0.26	2	0.08	43	20.70
S24D	−12.7806	−0.1383	−4,453	0.25^a^			40^a^	
L25D	−13.2230	−0.1745	−4,207	0.24^a^			37^a^	
S26D	−13.6260	−0.3434	−4,216	0.23	2	0.01	35	3.05
L27A	−13.9427	−0.4007	−3,928	0.21^a^			32^a^	
I28D	−14.2684	−0.4918	−3,711	0.20	3	0.05	29	8.59
S29D	−14.6272	−0.5597	−3,626	0.23	3	0.10	37	24.20
L30A	−14.9467	−0.5880	−4,003	0.17	4	0.09	25	18.32
S31D	−15.3187	−0.7141	−3,408	0.18	5	0.11	28	22.10
S32D	−15.6470	−0.7968	−2,967	0.12^a^			18^a^	
L33D	−16.0152	−0.8747	−3,933	0.06	3	0.02	7	2.38
I34D	−16.3485	−0.9579	−2,964	0.10	2	0.08	14	11.24
S35D	−16.6798	−1.0372	−3,773					
I36D	−17.0306	−1.1170	−3,938					
L37D	−14.9718	1.5657	−5,054					
S38D	−12.7623	1.9218	−4,926	0.37	6	0.09	82	34.93
L39D	−11.4904	2.0557	−4,685					

*Note*. PI‐LAB = Passive Imaging of the Lithosphere‐Asthenosphere Boundary.

^a^ Linear interpolated values. NoE: Number of events.

## 3 Sediment Thickness Along the Equatorial MAR

The analysis of *P* and *P*_*d*_ *s* sediment phases using the PI‐LAB network gives a broad coverage over more than 1,000 km across the equatorial MAR, from 0.3 Myr old to 70.9 Myr (Figure 1). The delay time between the two phases shows a clear progression with depth and age starting with an average of 0.04 s at the ridge increasing to an average of 0.37 s (Table 1). The migrated sediment thicknesses are in the range of 5–82 m and increase with age and depth (Figure 3).

In another approach, we confirmed the thickness of the sediments by analyzing the subbottom profiler data recorded from the onboard Knudsen echo sounder. In principle the sounder is used to determine the depth of the seafloor; however, subsurface reflections from sedimentary layers are sometimes visible (supporting information Figure S4). For example, the two‐way travel time profile for station L13D shows a sediment delay time of about 0.035 s between the upper and lower sediment interfaces yielding a thickness of about 54–64 m for *V*_*P*_ of 1.55–1.84 km/s, respectively, within the error of the *P*‐to‐*S* delay sediment thickness (50 ± 30 m, Table 1). Interestingly, the thicknesses determined from these two different, independent techniques are both smaller than the global model. Unfortunately, we only had Knudsen data available at a few sites to do this kind of comparison.

Our results agree well with the global total‐sediment‐thickness model of Whittaker et al. (2013) up to about 15–20 Myr, after which, our sediment thicknesses are significantly less than the global model (Figures 3a–3c). A similar *P*‐to‐*S* study of sediment thickness on the 0‐ to 10‐Myr‐old Juan de Fuca and Gorda Plates (Rychert et al., 2018) also found a one‐to‐one trend with the global model except for beneath the continental margin, where *P*‐to‐*S* thicknesses were greater than the global model, but in agreement with refraction work (Horning et al., 2016; Figure 3b, inset chart).

In general, the sediment thickness increases with age but our results show a clear change in slope, steeper <10 Myr closer to the ridge, and less steep on the older seafloor (Figure 3d). The slopes are a long‐term proxy for sedimentation rate. We determine a sediment deposition rate that fits the data, taking into consideration the compaction of sediments over time. We adopt the relationship for decompaction determined for the Madeira Abyssal Plain in the North Atlantic by Alibés et al. (1996). They studied various lithologic layers using data acquired from seismic reflection lines and borehole data and found a compaction of approximately 40% of the original thickness, represented by

$$\mathrm{decompacted}\;\mathrm{depth}=0.6974\times \mathrm{compacted}\;\mathrm{dept}h^{\left(1.1507\right)}$$

(Alibés et al., 1996; Alibés et al., 1999). We find our compacted sediment thickness can be fit with a sediment deposition rate of 5 mm/kyr for young seafloor (<10 Myr), and a deposition rate of 1.2 mm/kyr for seafloor older than 10 Myr (Figure 3d). We also find that sediment thickness is symmetric across both sides of the ridge, with the two sedimentation rates applicable to either side, at least up to 25 Myr (Figure 3d). This means that both flanks have undergone the same sedimentation process.

Our results can be compared with direct sediment analysis from IODP cores drilled on the eastern side of the ridge in seafloor 3.7 Myr old, close to our southern profile (Figure 1), and box cores throughout the region (Ku et al., 1968). The IODP cores sampled sediments to 200 meters depth (Ruddiman & Janecek, 1989) much deeper than our 15‐m result from nearby station S11D, which is about 65 and 84 km away. The very thick sediments were attributed to the fact that the sites were located in local basins close to the ridge, in an area characterized by strong topographic variations in the range of ∼1–3 km at very short distances <10–20 km (Harmon et al., 2018), receiving more sediment than the surrounding topographic highs with several slump/turbidity current events visible in more than 50% of the section (Ruddiman & Janecek, 1989). They propose a local deposition rate between 30 and 50 mm/kyr, which included the additional depositional events. Our deposition rate of 1.2 mm/kyr, however, match closely with rates derived from the ionium age from box cores sites close to our study region (0.8–1.5 mm/kyr; Ku et al., 1968).

Agreement with the global model of sediment thickness up to 15–20 Myr suggests that the large increase in sedimentation rate at 10 Myr is likely encompassed in the global model. Our thinner sediments on seafloor older than 15–20 Myr could be at least in part related to the selection of the sites which were targeted for local topographic highs (Figure 3c). Thirteen out of 21 stations are located on shallower bathymetry than predicted by half‐space cooling (Figure 3c). This means that our result probably represents a lower bound on sediment thickness and rate. An extreme example of this is S38D, which has a thickness of 82 m located on seafloor 70.9 Myr, much thinner than the 486‐m thickness found in the global model. The thin sediment at this location is also confirmed from the subbottom profiles (supporting information Figure S4). Such thin sediments are not uncommon and can be found elsewhere across the Atlantic (e.g., Ewing & Ewing, 1967; Mehouachi & Singh, 2018). This station is located on a regional topographic high, whereas the global model is likely influenced by terrestrial sediments coming off of Africa. Overall agreement of station S38D with the sediment deposition rates and trends found at all of our stations, and disagreement with the global model, suggests that our sediment deposition rates represent a lower limit, appropriate for topographic highs, in comparison to the global model which represents an upper limit appropriate for basins.

The observed change in the rate of deposition at 10 Myr is likely related to several important climatic changes that occurred 7–10 Ma, with even greater enhancement in the past 3 Ma, caused by increased glaciation (Ravelo et al., 2004). Specifically, data from marine sediment cores suggest that eolian dust fluxes from Africa to the Atlantic varied dramatically during the last 10 Myr (e.g., Ruddiman, Sarnthein, et al., 1989; Tiedemann et al., 1994). One contributing factor for the change in dust flux is tectonically driven large‐scale, rapid uplift during the latest Cenozoic (8–10 Myr) in East Africa, and South America (Ruddiman, Prell, et al., 1989) and farther away in Southeast Asia (Molnar et al., 1993), which affect climate patterns such as the African and Asian Monsoon. In addition, the shrinkage of the Tethys Sea during the Tortonian (7–11 Myr) could have affected the African summer monsoon (Zhang et al., 2014). In the last 3 Myr there has been a decrease in the North Atlantic sea surface temperatures (de Menocal, 1995), related to the gradual global cooling in the Pliocene epoch (3 Myr) resulting in regional climate shifts (Ravelo et al., 2004). These changes in climate and sea surface temperature affect the biogenic production rate as noted in the last few million years (e.g., Ruddiman & Janecek, 1989).

## 4 Conclusion

We determine the sediment thickness along the equatorial MAR inferred from delay times of *P*‐to‐*S* converted phases at the crust‐sediment boundary using teleseismic earthquakes recorded by the PI‐LAB seismic network. The average *P*_*d*_ *s*‐*P* delay times range from 0.04 to 0.37 s, which translates to 5‐ to 82‐m thickness across seafloor age from 0.3 to 70.9 Myr old.

Sediment thickness increases with age, as expected. Our thickness estimates are in agreement with the global model for young (<15–20 Myr old) seafloor but are substantially thinner at older ages (Whittaker et al., 2013). The discrepancy could be influenced by differences in site location; those of the ocean drilling measurements were located in basins, whereas ours are often on topographic highs. Our result may represent a lower limit and suggests the rate of sediment deposition decreases from 5 mm/kyr at about 10 Myr to 1.2 mm/kyr for older seafloor. The higher rate of recent deposition may be caused by changes in climate that caused higher flux of dust and/or biogenic activity.

## Supporting information



Supporting Information S1
