# Supplementary material for: Sediment Characterization at the Equatorial Mid‐Atlantic Ridge From P‐to‐S Teleseismic Phase Conversions Recorded on the PI‐LAB Experiment
Source: Geophys Res Lett. 2018 Nov 23;45(22):12244–52. doi: 10.1029/2018GL080565 (PMC6360481; doi:10.1029/2018GL080565)
Supplement: Supplementary file 1 — Supporting Information S1 [file GRL-45-12244-s001.pdf]

Supporting Information for "Sediment characterization at the equatorial Mid-Atlantic Ridge from *P*-to-*S* teleseismic phase conversions recorded on the PI-LAB experiment"

M. R. Agius[1,2], N. Harmon[1], C. A. Rychert[1], S. Tharimena[1,3], and J.-M. Kendall[4]

[1]Ocean and Earth Science, University of Southampton, United Kingdom.

[2]Now at the Department of Geosciences, Faculty of Science, University of Malta, Malta.

[3]Now at Jet Propulsion Laboratory, California Institute of Technology, Pasadena, CA, USA.

[4]School of Earth Sciences, University of Bristol, United Kingdom.

Corresponding author: Matthew R. Agius, `matthew.agius@soton.ac.uk`

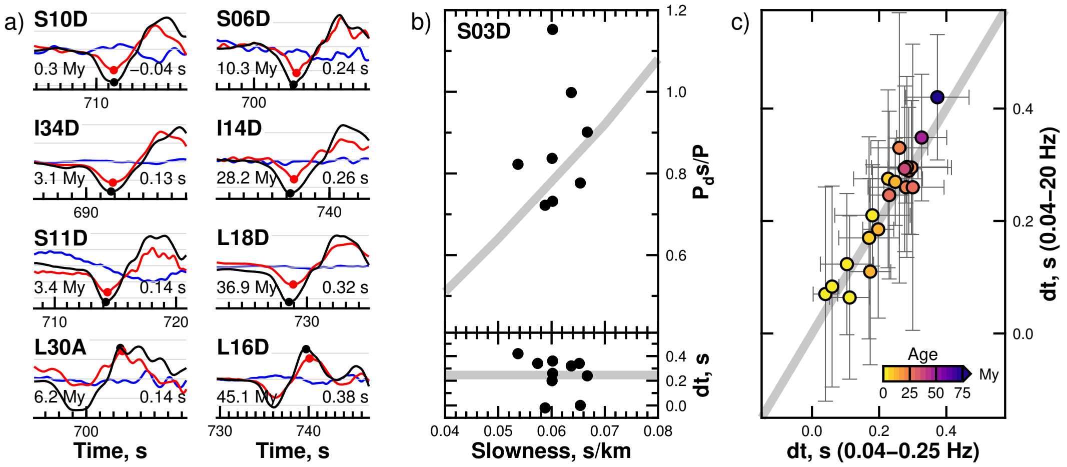

Figure 1. Data analysed using high-pass filter 0.04–20 Hz. Examples of Ps sediment conversions, relationship of P_ds/P amplitude ratio and delay time (P_ds-P, dt) with respect to slowness, and a comparison between the delay times using two different filter ranges a) Black and red waveforms show the direct P wave and the converted S wave on the vertical and radial component, respectively. Blue seismogram is the transverse component. The maximum absolute amplitude of the P phase and the following S phase are marked with dots. The seafloor age and delay time are indicated in the bottom left and right, respectively. Each frame is the record from a different station for the same teleseismic earthquake (24/11/2016 18:43 UTC, Mw 7.0). b) (Top) P_ds/P amplitude ratio with respect to slowness for earthquakes recorded at station S03D. Gray line: P_ds/P ratio from synthetic waveforms using a 1-D model and a range of ray parameters. (Bottom) Delay time with respect to slowness for earthquakes recorded at station S03D. Thick, horizontal gray line: Average Ps delay time for the station. c) Dot shows the stations' delay time for two different filters (0.04–0.25 Hz and 0.04–20 Hz) and their respective error bars. Coloured dots represent the seafloor age beneath the respective station. Gray line represents the one-to-one relationship between the two filters

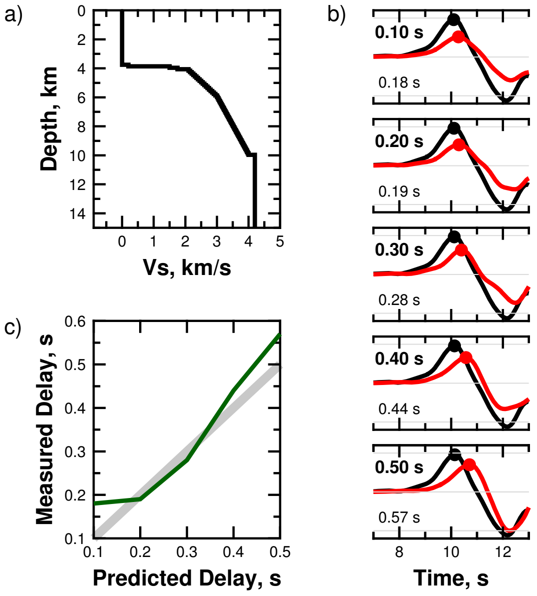

Figure 2. Synthetic tests for sedimentary delay time measurements. A series of 1-D velocity models (a) with varying sedimentary thickness are generated to represent the range of sediment delay times (0.1–0.5 seconds, b). The peak in the synthetic black and red waveforms [*Shearer and Orcutt*, 1987] represent the P phase and the sedimentary P-to-S (P_ds) converted phase on the vertical and radial component, respectively. The measured delay times are shown in the bottom left corner of each frame. c) Comparison between the predicted (green line) and the measured delay time.

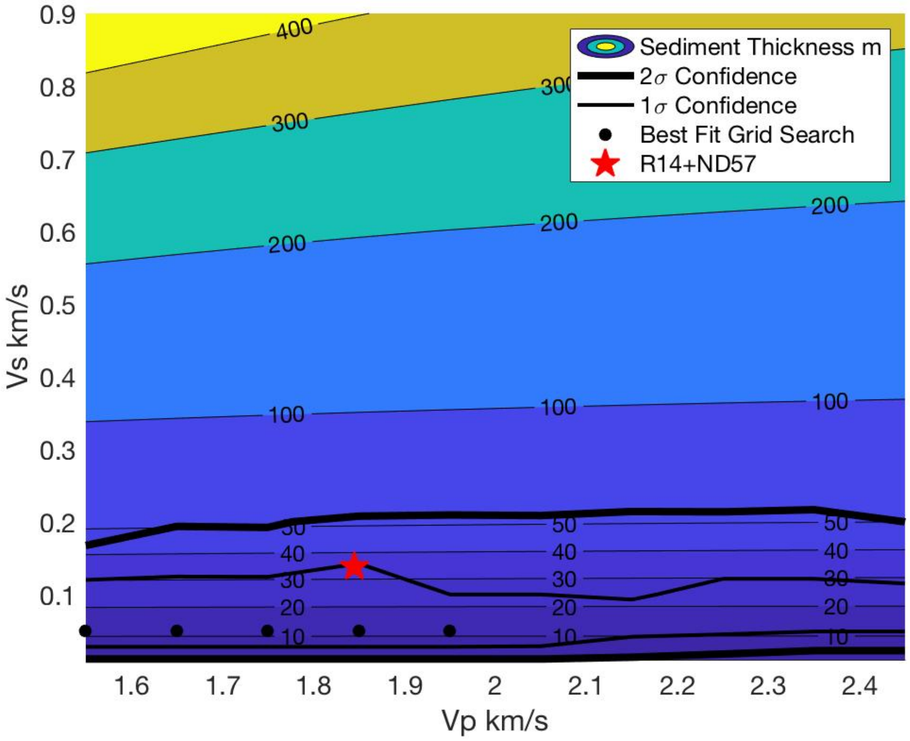

Figure 3. Grid search over V_P and V_S at example station S03D, fitting both the amplitudes and delay times of synthetics to that of data. Corresponding sediment thicknesses are contoured in the background. The average delay time of station S03D is 0.23 seconds. A 10 s period tapered sine wave is used as a synthetic source. Thin and thick contour plots show the 1σ and 2σ confidence, respectively. Black dots represent best fits. Red star represents the sediment thickness derived from *Ruan et al.* [2014] (R14) and *Nafe and Drake* [1957] (ND57) sediment thickness-velocity relationships, used here. Lack of sensitivity to P wave is due to the order of magnitude difference between the P- and S-wave velocity, so the delay time is dominated by the shear velocity vertical slowness, especially for thin sediments. In addition, the amplitude of the conversion is highly sensitive to the shear velocity.

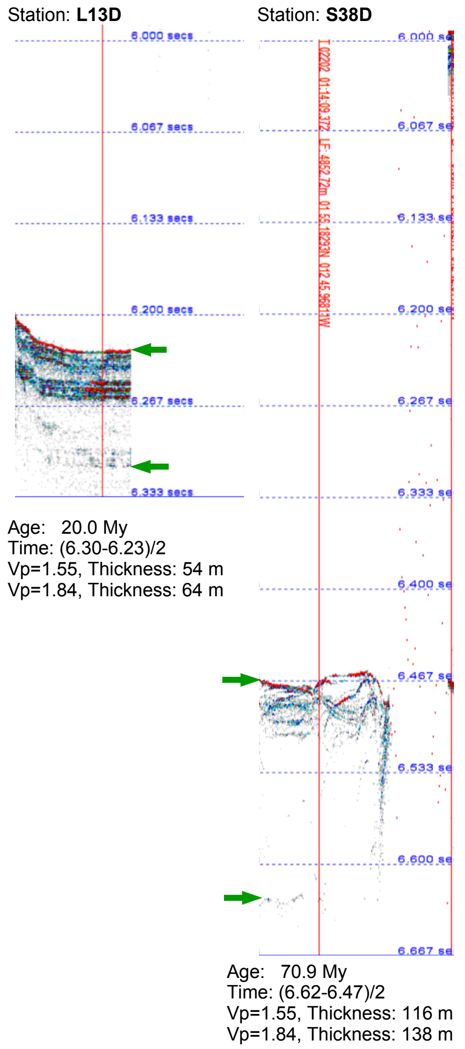

Figure 4. Examples of two-way-travel time recorded during station deployment using Knudsen Chirp Echosounder. Green arrows mark the selected upper and lower sediment interface. Station S38D is located in deeper water than L13D. Thicknesses are calculated using velocities ~1.55 km/s obtained from nearby cores (IODP sites 662 and 663, *Ruddiman et al.* [1988a,b]) and V_P=1.84 km/s obtained from reflection [*Nafe and Drake*, 1957].

References

Nafe, J. E., and C. L. Drake (1957), Variation with depth in shallow and deep water marine sediments of porosity, density and the velocities of compressional and shear waves, *Geophysics*, *22*(3), 523, doi:10.1190/1.1438386.

Ruan, Y., D. W. Forsyth, and S. W. Bell (2014), Marine sediment shear velocity structure from the ratio of displacement to pressure of Rayleigh waves at seafloor, *J. Geophys. Res.*, *119*(8), doi:10.1002/2014JB011162.

Ruddiman, W., M. Sarnthein, J. Baldauf, and S. S. Party (1988a), Proceedings Initial Reports, 7. Site 662, in *Proceedings of the Ocean Drilling Program, 108 Scientific Results*, vol. 108, Ocean Drilling Program.

Ruddiman, W., M. Sarnthein, J. Baldauf, and S. S. Party (1988b), Proceedings Initial Reports, 8. Site 663, in *Proceedings of the Ocean Drilling Program, 108 Scientific Results*, vol. 108, Ocean Drilling Program.

Shearer, P. M., and J. A. Orcutt (1987), Surface and near-surface effects on seismic waves—theory and borehole seismometer results, *Bull. Seismol. Soc. Am.*, *77*(4), 1168.
